# Thresholds for Arterial Wall Inflammation Quantified by ^18^F-FDG PET Imaging: Implications for Vascular Interventional Studies

**DOI:** 10.1016/j.jcmg.2016.04.007

**Published:** 2016-10

**Authors:** Fleur M. van der Valk, Simone L. Verweij, Koos A.H. Zwinderman, Aart C. Strang, Yannick Kaiser, Henk A. Marquering, Aart J. Nederveen, Erik S.G. Stroes, Hein J. Verberne, James H.F. Rudd

**Affiliations:** a Department of Vascular Medicine, Academic Medical Centre, Amsterdam, the Netherlands; b Department of Clinical Epidemiology, Academic Medical Center, Amsterdam, the Netherlands; c Department of Radiology, Academic Medical Center, Amsterdam, the Netherlands; d Biomedical Engineering and Physics, Academic Medical Center, Amsterdam, the Netherlands; e Department of Nuclear Medicine, Academic Medical Center, Amsterdam, the Netherlands; f Division of Cardiovascular Medicine, University of Cambridge, Cambridge, United Kingdom

**Keywords:** ^18^F-FDG PET/CT, atherosclerosis, imaging, inflammation, thresholds, %_active slices_, percentage of active slices, CT, computed tomography, CVD, cardiovascular disease, ^18^F-FDG, ^18^fluorodeoxyglucose, ICC, intraclass coefficient correlation, PET, positron emission tomography, ROI, region of interest, SUV, standardized uptake value, SUV_max_, maximum standardized uptake value, TBR, target to background ratio, TBR_active slices_, percentage having at least 1 active slice, TBR_max_, 90th percentile of target to background ratio

## Abstract

**Objectives:**

This study assessed 5 frequently applied arterial ^18^fluorodeoxyglucose (^18^F-FDG) uptake metrics in healthy control subjects, those with risk factors and patients with cardiovascular disease (CVD), to derive uptake thresholds in each subject group. Additionally, we tested the reproducibility of these measures and produced recommended sample sizes for interventional drug studies.

**Background:**

^18^F-FDG positron emission tomography (PET) can identify plaque inflammation as a surrogate endpoint for vascular interventional drug trials. However, an overview of ^18^F-FDG uptake metrics, threshold values, and reproducibility in healthy compared with diseased subjects is not available.

**Methods:**

^18^F-FDG PET/CT of the carotid arteries and ascending aorta was performed in 83 subjects (61 ± 8 years) comprising 3 groups: 25 healthy controls, 23 patients at increased CVD risk, and 35 patients with known CVD. We quantified ^18^F-FDG uptake across the whole artery, the most-diseased segment, and within all active segments over several pre-defined cutoffs. We report these data with and without background corrections. Finally, we determined measurement reproducibility and recommended sample sizes for future drug studies based on these results.

**Results:**

All ^18^F-FDG uptake metrics were significantly different between healthy and diseased subjects for both the carotids and aorta. Thresholds of physiological ^18^F-FDG uptake were derived from healthy controls using the 90th percentile of their target to background ratio (TBR) value (TBR_max_); whole artery TBR_max_ is 1.84 for the carotids and 2.68 in the aorta. These were exceeded by >52% of risk factor patients and >67% of CVD patients. Reproducibility was excellent in all study groups (intraclass correlation coefficient >0.95). Using carotid TBR_max_ as a primary endpoint resulted in sample size estimates approximately 20% lower than aorta.

**Conclusions:**

We report thresholds for physiological ^18^F-FDG uptake in the arterial wall in healthy subjects, which are exceeded by the majority of CVD patients. This remains true, independent of readout vessel, signal quantification method, or the use of background correction. We also confirm the high reproducibility of ^18^F-FDG PET measures of inflammation. Nevertheless, because of overlap between subject categories and the relatively small population studied, these data have limited generalizability until substantiated in larger, prospective event-driven studies. (Vascular Inflammation in Patients at Risk for Atherosclerotic Disease; NTR5006)

## Graphical abstract

**Figure:**
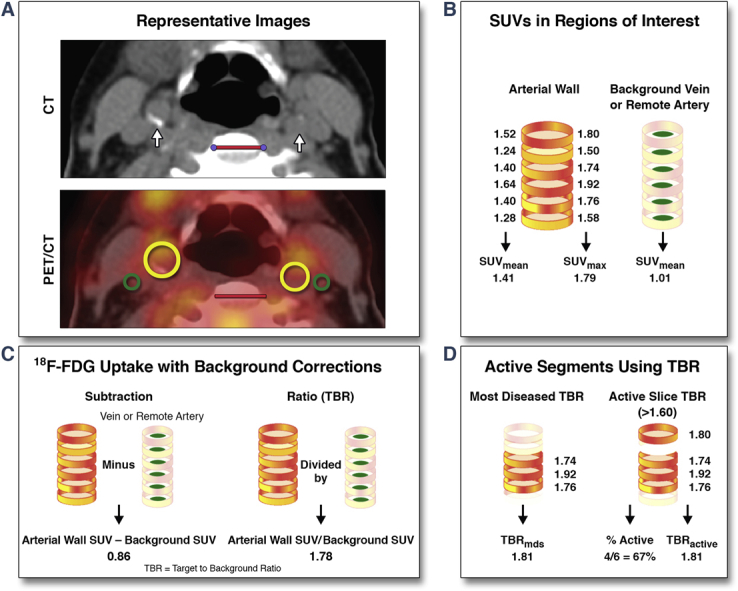

Atherosclerosis is a chronic, low-grade inflammatory disease of the arterial wall that causes myocardial infarction and stroke (1). Despite aggressive primary and secondary prevention strategies, long-term disability and death from cardiovascular disease (CVD) continue to increase (2). Arterial inflammation is strongly related to the risk of atherosclerotic plaque rupture. Quantification of inflammation may improve patient risk stratification and allow new drug therapies to be tested (1).

Noninvasive imaging, in particular with ^18^F-fluordeoxyglucose (^18^F-FDG) positron emission tomography (PET), has been used in this way 3, 4. Arterial wall ^18^F-FDG uptake mirrors inflammatory activity in atherosclerosis 5, 6, 7; inflammatory cells consume large amounts of glucose in comparison with other plaque cells. This results in ^18^F-FDG accumulation. In addition, arterial ^18^F-FDG uptake is higher in morphologically unstable plaques and predicts future vascular events 8, 9, 10, 11, 12, 13.

^18^F-FDG PET can assess the efficacy (or futility) of treatments designed to lower plaque inflammation 14, 15, 16, 17, 18, 19, 20, 21, 22, 23, 24, 25, 26, 27. As shown in Online Table 1, the number of vascular intervention trials using ^18^F-FDG PET as a surrogate marker of inflammation is growing, with one-half being published in the past 2 years. Several of these studies enriched their study populations by excluding subjects with ^18^F-FDG uptake below pre-defined thresholds. However, a consensus regarding the most appropriate thresholds is lacking 28, 29, 30, 31, primarily because healthy subjects, presumably without pathological arterial inflammation, have not been systematically imaged, and large-scale prospective outcome studies are awaited 32, 33. Without these data, it is challenging to enroll patients with sufficient arterial inflammation to need therapy and to avoid randomizing those unlikely to respond.

In this study, we assessed 5 frequently applied arterial ^18^F-FDG uptake metrics in 3 distinct groups: healthy control subjects, those with risk factors for CVD, and a group with established CVD. Considering ^18^F-FDG uptake in the arterial wall of healthy control subjects as physiological, we determined the 90th percentile for arterial wall inflammatory activity using several commonly reported PET endpoints. Finally, we determined the reproducibility of published measures of ^18^F-FDG uptake and derived optimal sample sizes for drug studies based on our results.

## Methods

### Study population

We recruited subjects into 3 groups: 1) healthy control subjects; 2) patients at increased CVD risk (Framingham risk score >10%); and 3) patients with known CVD (experienced myocardial infarction, transient ischemic attack, stroke, or carotid artery atherosclerosis >12 months before PET imaging). Healthy control subjects were recruited via advertisements in newspapers and screened to exclude those with a history of CVD, cardiovascular risk factors, or medication use. All healthy control subjects had a value of 0 for coronary artery calcium score. Exclusion criteria for all subjects were age <40 years, diabetes mellitus, or inflammatory or malignant disease. ^18^F-FDG PET/computed tomography (CT) imaging was performed at the Academic Medical Center, Amsterdam, the Netherlands. Ten subjects underwent repeated imaging after 3 weeks to assess interscan reproducibility. All subjects provided written informed consent. The study was approved by the local institutional review board and conducted according to the principles of the International Conference on Harmonization–Good Clinical Practice.

### Biometric and biochemical measurements

Presence of cardiovascular risk factors and use of medication were assessed by questionnaire. EDTA plasma was obtained to measure total cholesterol, high-density lipoprotein cholesterol, triglycerides, C-reactive protein, glucose, creatinine, and leukocyte and monocyte counts using commercially available enzymatic methods. Low-density lipoprotein cholesterol levels were calculated using the Friedewald equation.

### ^18^F-FDG PET/CT imaging and analysis

^18^F-FDG PET/CT imaging was performed on a PET/CT scanner (Philips, Best, the Netherlands). Subjects fasted for >6 h before infusion of 200 MBq of ^18^F-FDG (5.5 mCi). PET imaging was initiated with a low-dose, non–contrast-enhanced CT for attenuation correction and anatomic co-registration (slice thickness 3 mm) 90 min after ^18^F-FDG administration. Additionally, CT scans were used for coronary artery calcium scoring (Online Appendix). Images were analyzed using OsiriX software (Geneva, Switzerland).

**Figure 1:**
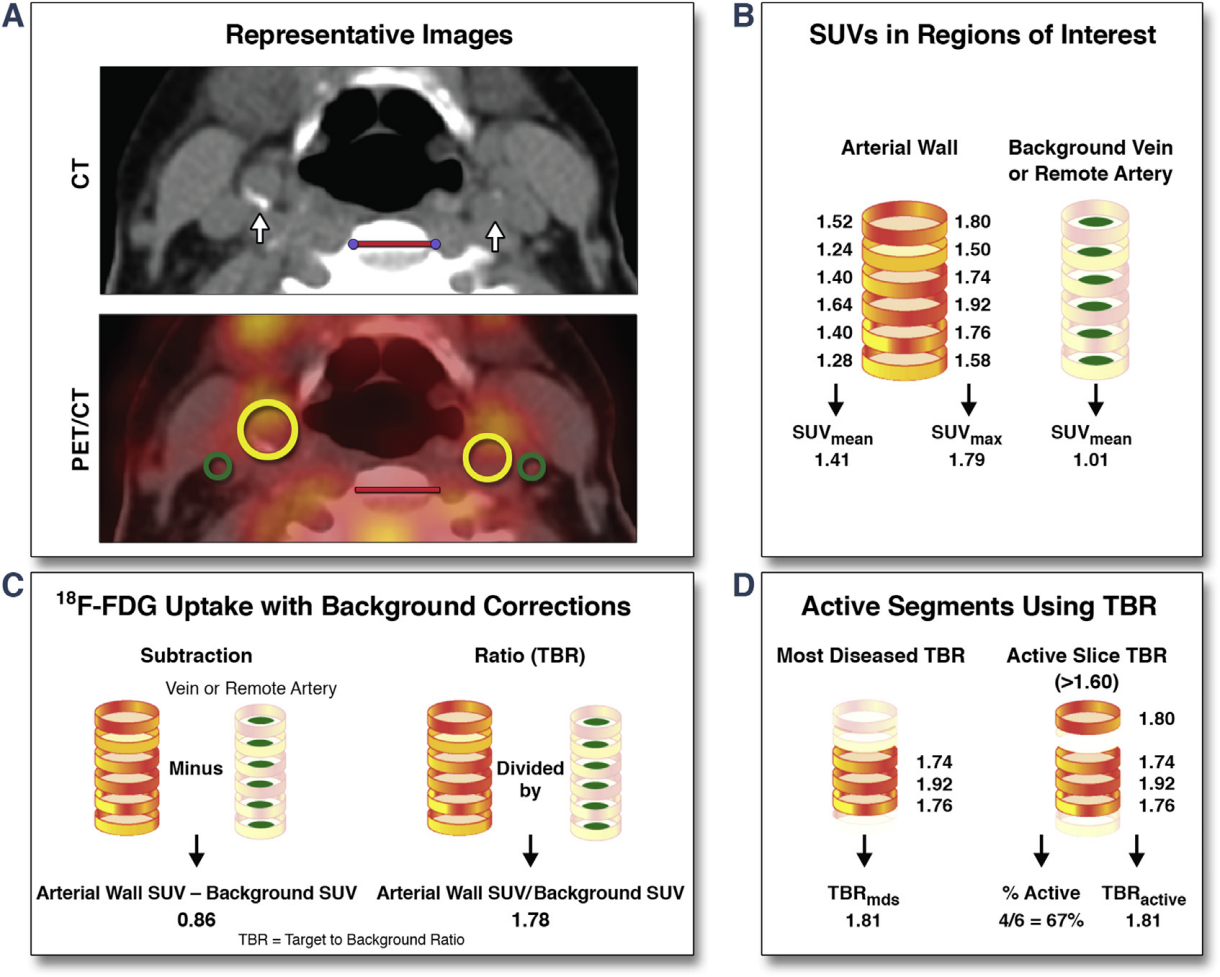
Arterial PET/CT Images and Analysis Methods **(A)** Representative CT and ^18^F-FDG PET/CT images of the carotid arteries **(white arrow, yellow ROIs)** and jugular veins **(green ROIs)** in a patient with cardiovascular disease. **Red scale bars** indicate 2 cm. Schematics showing **(B)** standardized uptake values (SUVs) in the whole artery and the background, **(C)** background corrections, and **(D)** active segment analysis with corresponding imaging parameters. A similar analysis is performed for the aortic segment. CT = computed tomography; ^18^F-FDG = ^18^fluorodeoxyglucose; MDS = most diseased segment; PET = positron emission tomography; ROI = regions of interest; SUV_max_ = maximum standardized uptake value; SUV_mean_ = mean standardized uptake value; TBR = target to background ratio.

Figure 1 provides an overview of the ^18^F-FDG uptake analysis and metrics. ^18^F-FDG uptake was assessed in: 1) the carotids starting from 1 slice caudal to the carotid bifurcation downwards; and 2) in the aorta from 1 slice cranial to the pulmonary arteries upwards, per standard methods (34). From each region of interest (ROI), standardized uptake values (SUVs) were read. SUV represents ^18^F-FDG activity adjusted for ^18^F-FDG dose, corrected for decay, and divided by body weight. To correct for background ^18^F-FDG, whole artery SUV was either subtracted or divided (target to background ratio [TBR]) by background SUV obtained from venous or remote arterial blood. After whole artery metrics, the most-diseased segment TBR was recorded as the mean of 3 adjacent slices with the highest arterial maximum standardized uptake value (SUV_max_). In the active segment analysis, slices with 90th percentile of their TBR (TBR_max_) values above a pre-defined cutoff level (either ≥1.60, ≥1.80, or ≥2.00 for the carotid arteries; ≥2.40, ≥2.60, or ≥2.80 for the aorta) were considered active, whereas noninflamed segments were excluded. Using this approach, the percentage of those having at least 1 active slice (TBR_active slices_), the TBR_active slices_ and the percentage of active slices (%_active slices_) were assessed.

### Statistical analysis

Continuous variables are expressed as mean ± SD or median and interquartile range, unless otherwise specified. Differences in ^18^F-FDG uptake between the different groups were assessed using a multivariate model to account for age, sex, hypertension (systolic blood pressure >140 mm Hg, diastolic blood pressure >90 mm Hg, or use of antihypertensive medication), body mass index, smoking, drug use (statins, ezetimibe, angiotensin-converting enzyme inhibitors, acetylsalicylic acid, beta-blockers), lipid profile, and glucose. We estimated SUV and TBR upper threshold values based on the tolerance interval (35) using the 95th and 90th percentiles of log-normal SUV and TBR in the healthy control subjects.

Power analyses to detect the superiority of a test over a control in SUV and TBR were based on a 2-sample unpaired Student *t* test (2-sided) and performed with 80% power and an alpha of 5%. The agreement between scans and analyses were assessed using intraclass correlation coefficients (ICC, r) and Bland-Altman plots. The SD of the paired differences and the coefficient of variation between the initial and repeat scans were calculated. Coefficient of variation was calculated by dividing the SD of the paired differences by the mean value of the population for each parameter. Values of p < 0.05 were considered statistically significant. Data were analyzed using SPSS version 19.0 (SPSS Inc., Chicago, Illinois).

## Results

### Clinical characteristics

In total, 83 participants (61 ± 8 years of age) were imaged, including 25 healthy control subjects, 23 patients at increased CVD risk (median Framingham score 14% [interquartile range: 4]), and 35 patients with a history of CVD documented as significant carotid artery stenosis (n = 13), transient ischemic attack (n = 9), stroke (n = 9), and/or myocardial infarction (n = 25). Subject demographics are listed in Table 1.

### Whole artery ^18^F-FDG uptake

Whole artery ^18^F-FDG in the carotids and aorta, expressed as SUV_max_, showed a gradual increase from healthy to diseased subjects (Table 2). The mean difference in SUV_max_ between healthy control subjects and those at increased CVD risk was 0.30 ± 0.08 for the carotids and 0.36 ± 0.09 for the aorta. The mean difference in SUV_max_ between patients at increased CVD risk and patients with known CVD was 0.10 ± 0.08 for the carotids and 0.28 ± 0.10 for the aorta.

Before calculating subtraction or ratio metrics, we demonstrated that both venous and arterial blood ^18^F-FDG background values were comparable between groups (Table 2, Online Table 2). In line with this observation, ^18^F-FDG background corrections of the SUV values with either subtraction or ratio (TBR) did not affect the significance between groups (Table 2, Online Table 2).

### Active segment approach

We also examined the TBR of the most-diseased segment TBR (Online Table 2). In addition, an active segment analysis was performed using several pre-defined cutoffs. Using a cutoff of TBR ≥1.60 for the carotids, 48% of the healthy control subjects had at least 1 active slice compared with 96% and 100% of the patients at increased risk for or with known CVD, respectively (Table 2). The percentage of active slices was 32 ± 40% in healthy control subjects, 80 ± 31% in patients at risk for CVD, and 90 ± 19% in known CVD patients (p = 0.020). The corresponding TBR_active slices_ values were also distinct between groups (p = 0.044) (Table 2). With cutoffs of ≥1.80 or ≥2.00, the number of healthy control subjects with at least 1 active slice in the carotids decreased substantially (Online Table 3). Whereas the %_active slices_ remained significantly different between groups, the TBR_active slices_ did not (Online Table 3).

In contrast to the carotids, a much larger proportion of the subjects had active aortic walls. With a cutoff of ≥2.40, 88% of the healthy control subjects had at least 1 active slice; however, the TBR_active slices_ and %_active slices_ were not distinct between groups (Table 2). With the active definition at ≥2.60 or ≥2.80, more than one-half of the healthy control subjects still had active segments (Online Table 3). For the highest cutoff, TBR_active slices_ was significantly different between groups (p = 0.015).

### Thresholds

**Figure 2:**
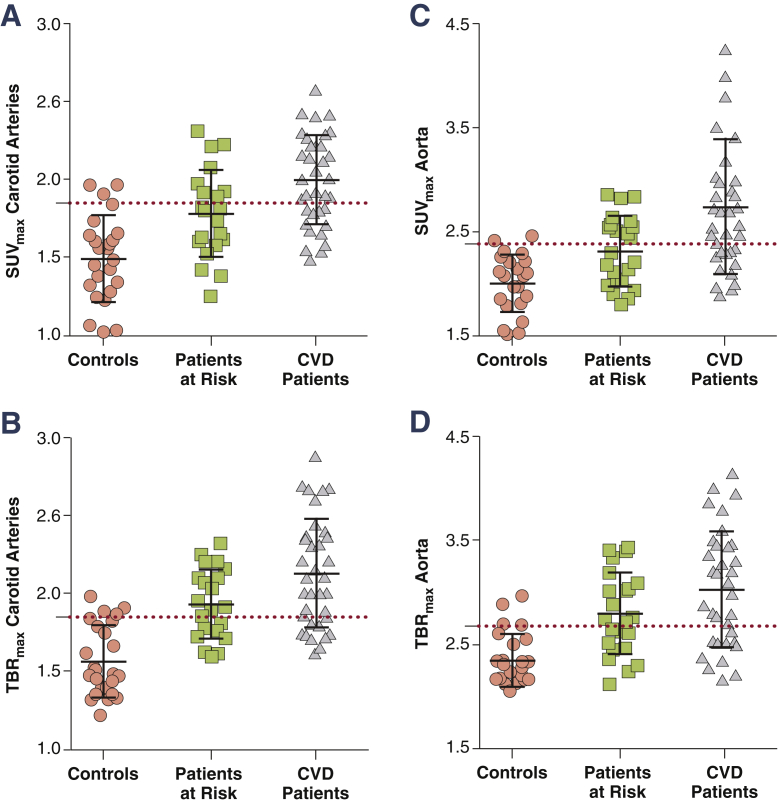
Gradual Increase of TBR_max_ in the Carotids and Aorta Between Groups Scatterplots showing the gradual increase in SUV_max_ and TBR_max_ for the carotids **(A and B)** and aorta **(C and D)** in healthy control subjects, patients at CVD risk and patients with known CVD. The red dashed line represents the 90th percentile value in healthy control subjects. CVD = cardiovascular disease; TBR_max_ = maximum target to background ratio; other abbreviations as in Figure 1.

The TBR thresholds based on the tolerance interval in healthy control subjects are listed in Table 3. Based on the 90th percentile of this interval, the threshold for SUV_max_ was 1.85 for the carotids and 2.38 for the aorta. For TBR_max_, this threshold was set at 1.84 for the carotids and 2.68 for the aorta. Figure 2 illustrates both the SUV_max_ and TBR_max_ values per group, with corresponding thresholds (red dashed lines). For SUV_max_, 39% to 43% of those at increased CVD risk versus 66% of the CVD patients exceeded these thresholds. For TBR_max_, these numbers were in general larger; 52% to 57% of those at increased CVD risk and 67% to 74% of CVD patients. In Online Table 4, we also provide the thresholds using the 95th percentile values.

### Sample sizes

**Figure 3:**
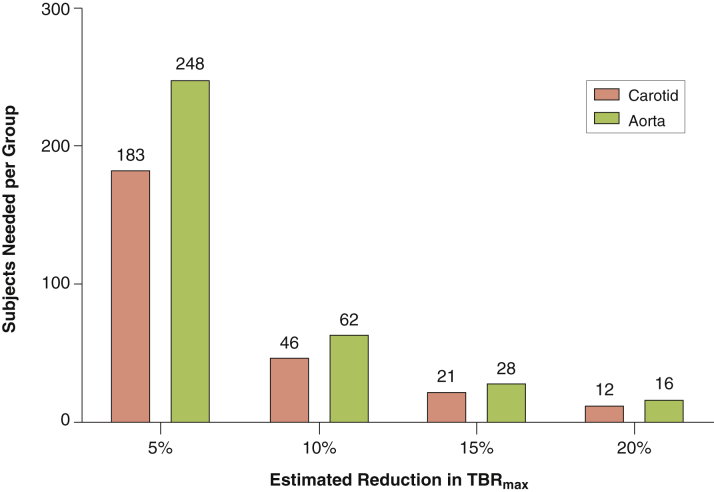
Estimated Sample Sizes for Vascular Intervention Studies Based on Our Results Sample sizes required for studies using TBR_max_ as the primary endpoint. These are dependent on the estimated drug effect (ranging between 5% and 20%) and target vessel for imaging (carotid artery or aorta). Abbreviation as in Figure 2.

Based on the TBR_max_ values in the present study, Figure 3 depicts the sample sizes required for an estimated drug effect; ranging from 5% to 20%, as has been observed in previous drug trials (Online Table 1). Carotid TBR as a primary endpoint requires approximately 20% fewer subjects compared with aorta TBR. Of note, sample sizes based on SUV_max_ values necessitate approximately 20% to 45% more subjects compared with TBR_max_ (Online Figure 1).

### Reproducibility

The intraobserver and interobserver and interscan agreement within 3 weeks was excellent for TBR_max_ as indicated by: 1) ICC values of >0.95 with narrow 95% confidence intervals; and 2) the absence of fixed or proportional bias in the Bland-Altman plots (Online Figure 2). In addition, agreement for all ^18^F-FDG metrics was also excellent in healthy control subjects (Online Tables 4 and 5).

## Discussion

In the present work, we tested 5 frequently applied approaches to quantify ^18^F-FDG uptake in the arterial wall of healthy controls, patients at risk, and patients with known CVD. Whole artery SUV_max_ was significantly different between groups, and ^18^F-FDG venous blood background values were similar. As such, ^18^F-FDG uptake metrics with background corrections, such as the subtraction or ratio method (TBR), remained significantly different. Moreover, the TBR and active slice methods accentuated differences between the groups. On the basis of these measures, we determined threshold values for arterial wall inflammation and found that >39% of patients at risk for and >66% with known CVD had inflamed arterial walls, highlighting a potential therapeutic window for additional anti-inflammatory strategies. Nevertheless, because of the substantial overlap between healthy controls and patients, the value of ^18^F-FDG PET for individual risk assessment is limited.

### ^18^F-FDG uptake metrics

Here, we assessed the most commonly reported ^18^F-FDG endpoints: 1) whole artery SUV; 2) background subtraction; 3) background ratio (TBR); 4) most-diseased segment; and 5) active segments. These different approaches highlight the ability of a single PET scan to measure multiple aspects of artery’s inflammatory status; by the same token, however, the use of multiple endpoints in drug studies is statistically less robust than a single readout (31).

As shown in Table 2, differences in background ^18^F-FDG activity between groups exist but are not significantly different. Both background correction methods show smaller variations compared with SUV; in patients with established CVD, the carotid SUV SD is 0.37 versus 0.28 to 0.30 after background correction. Consequently, the sample size based on TBR as readout is smaller than SUV. In addition, in drug studies with repeat imaging, the use of a ratio, such as TBR, limits the effect on signal quantification where variation between scans exists (e.g., weight change, ^18^F-FDG dose change, ^18^F-FDG circulation time change) (36). For these reasons, we favor the use of TBR, as also endorsed in the recent European Association of Nuclear Medicine position paper on vascular PET imaging (36).

With respect to the active segment approach, a substantial bias is induced by eliminating (a potentially large number of) included subjects and imaged slices (e.g., 48% of healthy subjects included in the carotid analysis). Consequently, the TBR_active slice_ loses much of its power to differentiate between healthy and diseased subjects. Hence, this approach should be interpreted with caution, and might be better suited for changes within 1 individual 20, 26.

### Inflammation in different arterial beds

The validation of ^18^F-FDG as a marker of plaque inflammation originates from histology 5, 6, 7 and gene expression studies 37, 38 performed on human carotid plaque material. Over time, quantification of ^18^F-FDG uptake in the aorta became adopted, supported by, among others, the histological work in rabbit models 39, 40 and the incremental value in cardiovascular risk stratification (11). The present study was not designed to investigate the nature of ^18^F-FDG vascular uptake, but nevertheless showed that SUVs and TBRs were consistently higher in the aorta compared with the carotids. This is relevant when applying an “index vessel approach” to drug trials because, in ∼80% of subjects, the index vessel will originate from the aorta (31). This might be suboptimal, as we also demonstrated that aortic TBR as endpoint requires a larger sample size to detect drug efficacy (37). Taking into account that the published drug-induced TBR changes have been relatively small (ranging between 5% and 15%) (Online Table 1), the optimal choice of endpoint vessel is important. The use of the carotid artery as a readout vessel holds the strongest biological validation linking the ^18^F-FDG signal and inflammation to recommend it 5, 6, 7, 13, 37, 38. Therefore, we suggest that if the index vessel approach is not used, the carotid artery is best validated as primary readout vessel, as highlighted by Gholami et al. (31).

### Thresholds for atherosclerotic inflammation

Previously, histological carotid plaque studies demonstrated the correlation between plaque rupture and inflammation 41, 42, 43; macrophage-rich areas in carotid plaques were higher in symptomatic patients (18 ± 10%) compared with asymptomatic patients (11 ± 4%) (42). Tawakol et al. (6) were the first to link plaque macrophages ex vivo to plaque inflammation in vivo, demonstrating a linear relation between macrophage content and ^18^F-FDG uptake in plaques of 17 patients scheduled for carotid endarterectomy. Carotid plaques with a macrophage area of <5% had low TBR values, whereas inflamed carotid plaques with macrophage areas >5% had carotid TBRs between 1.80 and 2.40 (25th and 75th percentiles) (6).

Instead of histology-based approaches, here we classified arterial wall inflammation using population-based data by regarding the 90th percentile of ^18^F-FDG uptake metrics in healthy controls as a natural threshold. Reassuringly, our healthy control data are consistent with ^18^F-FDG uptake values reported in prior studies 22, 44, 45, 46. In addition, our carotid uptake values are comparable to those reported in histology- 6, 41, 42, 47 and epidemiology-based (46) studies, further supporting the validity of our data.

### Reproducibility

In line with previous studies 34, 48, we report excellent reproducibility of PET atherosclerosis imaging in patients at risk and with known CVD, and extended the findings into healthy control subjects. We derived ICCs for interobserver variability of >0.95, similar to values reported previously 34, 48. Further, we document low interscan TBR changes (<3.5% over a 3-week period), which is in line with previous placebo-controlled intervention studies revealing small variations during a 3- to 6-month timeframe 19, 23. This makes PET/CT a highly reproducible and sensitive tool suitable for identifying patients for anti-inflammatory interventions and for determining their effectiveness.

### Study limitations

First, this limited observational study does not address the predictive value of arterial PET imaging. Using the present population-based approach, substantial overlap in ^18^F-FDG metrics between healthy and diseased subjects exists; therefore, ^18^F-FDG metrics should be correlated by outcome data to enable the assessment of “true” pathological ^18^F-FDG reference ranges in humans. For this, the results of larger, long-term prospective studies (BioImage [32] and Progression and Early detection of Subclinical Atherosclerosis [33]) are awaited. Second, despite the published recommendations on PET imaging protocols 34, 48, substantial variation in patient preparation (e.g., glucose levels, time of fasting), PET image protocol (e.g., time and areas of scanning) and technology (e.g., acquisition, reconstruction), and measurement parameters exists and harmonization is warranted 28, 29, 30, 31. As such, extrapolation of our thresholds is limited to studies using similar imaging and analysis protocols. Third, with respect to the population-based approach with a relative small group size, it must be stressed that clinical characteristics of the studied groups in this study (among others, age, sex, lipid levels) should be taken into account upon extrapolation of our thresholds. Finally, this study was not designed to associate ^18^F-FDG uptake with additional structural or functional features of the artery because we used a non–contrast-enhanced CT as part of the PET/CT. Future studies using magnetic resonance imaging should improve such assessments as well as correct for partial volume effects, which is a well-described limitation of PET imaging (31).

### Clinical relevance

For interventional studies, ^18^F-FDG PET can help to identify subgroups with inflammation above the physiological range and can provide reproducible measures of drug action. The majority of patients with known CVD have increased inflammatory activity in 1 or more arteries, despite standard-of-care treatments, including statin use in >80%. This residual inflammatory activity suggests the potential for further anti-inflammatory strategies in CVD patients (49). We await the results of large-scale studies of such interventions 50, 51. Nevertheless, because of the considerable overlap of ^18^F-FDG values between healthy control subjects, those at increased CVD risk, and patients with known CVD, it is uncertain whether ^18^F-FDG PET imaging is capable of identifying individual patients most likely to benefit from new therapies.

Perspectives **COMPETENCY IN MEDICAL KNOWLEDGE:** The majority of patients with CVD have increased inflammatory activity in 1 or more arteries, despite standard-of-care treatments, including statin use in >80%, reinforcing the potential room for additional anti-inflammatory strategies such as ^18^F-FDG PET. Arterial FDG uptake was assessed in healthy control subjects, those with risk factors, and patients with CVD to derive uptake thresholds in each subject group as well as the reproducibility of the measures.

**TRANSLATIONAL OUTLOOK:** Although the measured FDG metrics were reproducible and significantly different between healthy and diseased subjects, there was significant data overlap between subject categories limiting the generalizability of FDG PET until substantiated in larger, prospective event-driven studies.

## Figures and Tables

**Table 1:** Clinical Characteristics of Study Subjects

	Healthy Control Subjects (n = 25)	Patients at Increased CVD Risk (n = 23)	Patients With Known CVD (n = 35)	p Value∗	p Value†
Age, yrs	60 ± 11	59 ± 6	63 ± 7	NS	NS
Male	60 (15)	74 (17)	77 (27)	NS	NS
BMI, kg/m^2^	25 ± 3	26 ± 3	27 ± 4	NS	NS
SBP, mm Hg	134 ± 16	135 ± 9	133 ± 8	NS	NS
DBP, mm Hg	81 ± 10	82 ± 8	81 ± 7	NS	NS
Smoking	0 (0)	0 (0)	14 (5)	0.026	0.012
Lipid-lowering drugs, % yes	0 (0)	83 (19)	100 (35)	<0.001	NS
Statin use	0 (0)	83 (19)	86 (30)	<0.001	NS
Ezetimibe use	0 (0)	0 (0)	14 (5)	<0.001	<0.001
ACE inhibitor use	0 (0)	91 (21)	100 (35)	<0.001	NS
Acetylsalicylic acid use	0 (0)	70 (16)	100 (35)	<0.001	NS
Beta-blocker use, % yes	0 (0)	74 (17)	100 (35)	<0.001	NS
TChol, mmol/l	5.32 ± 0.96	7.33 ± 2.81	5.99 ± 3.16	0.040	NS
LDL-C, mmol/l	3.24 ± 0.97	5.42 ± 2.63	4.18 ± 3.11	0.011	NS
HDL-C, mmol/l	1.65 ± 0.37	1.21 ± 0.25	1.24 ± 0.37	<0.001	NS
TG, mmol/l	0.89 [0.84]	1.57 [0.99]	1.42 [0.91]	0.001	NS
Glucose, mmol/l	5.04 ± 0.33	5.40 ± 0.75	5.41 ± 1.19	NS	NS
Creatinine, μmol/l	79 [16]	80 [17]	82 [17]	NS	NS
Leukocytes, 10^9^/l	6.10 ± 1.74	6.30 ± 2.54	6.29 ± 1.52	NS	NS
Monocytes, 10^9^/l	0.45 ± 0.13	0.51 ± 0.16	0.54 ± 0.20	NS	NS
CRP, mg/l	1.30 [1.35]	1.20 [2.00]	2.30 [3.30]	NS	NS
CAC scores†‡	0 (0)	303 (110)	691 (372)	<0.001	<0.001

Values are mean ± SD, % (n), or median [IQR].

ACE = angiotensin-converting enzyme; BMI = body mass index; CAC score = coronary artery calcium score; CRP = C-reactive protein; DBP = diastolic blood pressure; HDL-C = high-density lipoprotein cholesterol; IQR = interquartile range; LDL-C = low-density lipoprotein cholesterol; NS = not significant; SBP = systolic blood pressure; TChol = total cholesterol; TG = triglycerides.

∗ p value between all groups.

† p value between patients at increased CVD risk and patients with known disease.

‡ Agatston score.

**Table 2:** Whole Artery and Active Segment Based ^18^F-FDG Uptake in Study Groups

	Healthy Control Subjects	Patients at Increased CVD Risk	Patients With Known CVD	p Value∗
Whole artery SUV_max_				
Carotid arteries	1.49 ± 0.28	1.79 ± 0.27	1.99 ± 0.37	<0.001
Ascending aorta	1.98 ± 0.31	2.34 ± 0.31	2.63 ± 0.63	<0.001
Venous background SUV_mean_				
Jugular veins	0.84 ± 0.13	0.92 ± 0.14	0.93 ± 0.18	NS
Superior vena cava	0.96 ± 0.11	0.84 ± 0.16	0.90 ± 0.20	NS
Arterial SUV–venous SUV_mean_				
Carotid arteries	0.53 ± 0.20	0.86 ± 0.22	0.96 ± 0.28	<0.001
Ascending aorta	1.14 ± 0.22	1.49 ± 0.23	1.73 ± 0.54	<0.001
Whole artery TBR_max_				
Carotid arteries	1.55 ± 0.23	1.94 ± 0.27	2.13 ± 0.30	<0.001
Ascending aorta	2.36 ± 0.25	2.80 ± 0.31	2.97 ± 0.59	<0.001
Active segment approach				
Carotid arteries (active ≥1.60)	48% of subjects	96% of patients	100% of patients	
% _active slices_	32 ± 40%	80 ± 31%	90 ± 19%	0.020
TBR_active slices_	1.79 ± 0.12	2.00 ± 0.29	2.09 ± 0.32	0.044
Ascending aorta (active ≥2.40)	88% of subjects	96% of patients	97% of patients	
%_active slices_	74 ± 30%	88 ± 25%	91 ± 18%	NS
TBR_active slices_	2.70 ± 0.21	2.97 ± 0.40	3.00 ± 0.49	NS

CVD = cardiovascular disease; ^18^F-FDG = ^18^fluorodeoxyglucose; SUV_max_ = maximum standardized uptake value; SUV_mean_ = mean standardized uptake value; TBR = target to background ratio (arterial wall SUV_max_ /venous background SUV_mean)_; TBR_max_ = 90th percentile of the TBR; TBR_active slices_ = percentage having at least 1 active slice.

∗ p Value for multivariate analysis adjusted for age, sex, hypertension, smoking, body mass index, drug usage, lipid profile, and glucose.

**Table 3:** ^18^F-FDG Uptake Threshold Values

Artery	Metric	Threshold∗	Percentage Above Threshold	
			Patients at Increased CVD Risk (%)	Patients With Known CVD (%)
Carotid	SUV_max_	>1.85	39	66
	TBR_max_	>1.84	52	74
Aorta	SUV_max_	>2.38	43	66
	TBR_max_	>2.68	57	67

Abbreviations as in Table 2.

∗ Thresholds were determined using the 90th percentile value observed in the healthy control subjects.
