# Supplementary material for: Thresholds for Arterial Wall Inflammation Quantified by 18F-FDG PET Imaging: Implications for Vascular Interventional Studies
Source: JACC Cardiovasc Imaging. 2016 Oct;9(10):1198–207. doi: 10.1016/j.jcmg.2016.04.007 (PMC5056585; doi:10.1016/j.jcmg.2016.04.007)
Supplement: Online Data [file mmc1.docx]

**ONLINE DATA SUPPLEMENT**

**Detailed methods**

*Coronary artery calcium score*

The CT scans were used to determine the Agatston score, the coronary artery calcium (CAC) score. In a manually set volume of interest, all pixels with intensity higher than 130 HU were selected. Connected areas of these threshold pixels were constructed. All areas smaller than 1 mm^2^ were excluded. The score was determined by combining all selected connected areas with a weight. The weight was determined by the highest intensity value of a pixel in the connected areas: 1 for 130–199 HU, 2 for 200–299 HU, 3 for 300–399 HU, and 4 for 400 HU and greater. Because of the difference in slice thickness of the images in his study (5 mm) compared to default Agatston score images (3mm), the sum CAC score was multiplied with 5/3.

**Online Table 1:** Previous intervention studies in patients with atherosclerosis using ^18^F-FDG PET/CT as surrogate endpoint

| **First author and reference** | **Study population** | **Arterial segments analyzed** | **PET as selection criterion** | **Intervention** | **Primary endpoint** | **Observed %change** |
| --- | --- | --- | --- | --- | --- | --- |
| Tahara et al. 2006(1) | 43 subjects with incidental arterial ^18^F-FDG uptake | Carotid and aorta combined | n.a. | Diet alone or with simvastatin (max 20 mg/day) for 3 months | SUV_max_ | -10% in diet+statin group |
| Lee et al. 2008(2) | 60 subjects with atherogenic risk factors | Carotid, subclavian, aorta and iliac arteries combined | n.a. | Life style modifications for 17 months | % positive lesions (SUV/blood pool >1) | -64.8% |
| Potter et al. 2009(3) | 30 post-stroke patients | Carotid, aorta and femoral arteries combined | n.a. | B-vitamin or placebo for 2 years | SUV_max_ | None |
| Ishii et al. 2010(4) | 30 patients with stable angina pectoris | Aorta, femoral | n.a. | Atorvastatin 5 or 20 mg/day for 6 months | TBR_mean_ | -7.9% in aortic, and  -9.9% in femoral TBR_mean_ in 20mg group |
| Wu et al. 2011(5) | 43 patients with atherosclerosis | Aorta and iliofemoral combined | n.a. | Atorvastatin 40 mg/day for 12 weeks | TBR_max_ | -19% |
| Fayad et al. 2011(6) | 130 patients with (risk factors for) coronary heart disease | Carotid, aorta | TBR_mds_ ≥1.60 | Dalcetrapib 600mg/day or placebo for 24 months | TBR_mds_ index vessel | None in TBR_mds_ index, whereas -7% in carotid TBR_mds_ |
| Elkhawad et al. 2012(7) | 99 patients with atherosclerosis | Carotid, aorta | n.a. | Losmapimod (p38MAPK inhibitor) 7.5 or 15 mg/day or placebo for 84 days | TBR_max_ index vessel | None in TBR_max_ index, whereas -5% in TBR_active segment_ index in low and high dose group |
| Tawakol et al. 2013(8) | 67 patients with (risk factors for) atherosclerosis | Carotid, aorta | TBR_mds_ ≥1.60 | Atorvastatin 10 or 80 mg/day for 3 months | TBR_mds_ index vessel | -14.42% in 80mg group |
| Van Wijk et al. 2014(9) | 24 patients with familial hypercholesterolemia | Carotid, aorta | n.a. | Lipid apheresis for 8 weeks | TBR_mds_ index vessel | -11.8% |
| Tawakol et al. 2014(10) | 71 patients with stable atherosclerosis | Carotid, aorta | n.a. | Rilapladib 250 mg/day or placebo for 3 months | TBR_max_ index vessel | None |
| Van der Valk et al. 2015(11) | 30 patients with atherosclerosis | Carotid | TBR_max_ ≥2.20 | 2 infusions with liposomal prednisolone or placebo, readout after 12 days | Carotid TBR_max_ | +7% |
| Kootte et al. 2015(12) | 7 patients with genetically determined low HDL | Carotid | n.a. | Infusions with apoA-I-containing HDL-mimetic for 6 months | TBR_max_ index vessel | -8.9% |
| Emami et al. 2015(13) | 72 patients with atherosclerosis | Carotid, aorta | TBR_max_ ≥1.60 | BMS-582949 (p38MAPK inhibitor) 100 mg/day, atorvastatin 80 mg/day or placebo for 12 weeks | TBR_max_ index vessel | None for BMS-582949, whereas -6% for atorvastatin group |
| Gaztanaga et al. 2016(14) | 52 patients with ACS 1-3 months, 45 completed | Carotid, aorta | n.a. | VIA-2291 (5-LO inhibitor) 100mg/day or placebo for 24 weeks | TBR_max_ index vessel | None |

HDL-c, high-density lipoprotein cholesterol; SUV, standardized uptake values; TBR, target to background ratio.

**References for Online Table 1**:

1. Tahara N, Kai H, Ishibashi M, et al. Simvastatin attenuates plaque inflammation: evaluation by fluorodeoxyglucose positron emission tomography. J. Am. Coll. Cardiol. 2006;48:1825–31.

2. Lee SJ, On YK, Lee EJ, Choi JY, Kim B-T, Lee K-H. Reversal of vascular 18F-FDG uptake with plasma high-density lipoprotein elevation by atherogenic risk reduction. J. Nucl. Med. 2008;49:1277–82.

3. Potter K, Lenzo N, Eikelboom JW, Arnolda LF, Beer C, Hankey GJ. Effect of long-term homocysteine reduction with B vitamins on arterial wall inflammation assessed by fluorodeoxyglucose positron emission tomography: a randomised double-blind, placebo-controlled trial. Cerebrovasc. Dis. 2009;27:259–65.

4. Ishii H, Nishio M, Takahashi H, et al. Comparison of atorvastatin 5 and 20 mg/d for reducing F-18 fluorodeoxyglucose uptake in atherosclerotic plaques on positron emission tomography/computed tomography: a randomized, investigator-blinded, open-label, 6-month study in Japanese adults scheduled. Clin. Ther. 2010;32:2337–47.

5. Wu Y-W, Kao H-L, Huang C-L, et al. The effects of 3-month atorvastatin therapy on arterial inflammation, calcification, abdominal adipose tissue and circulating biomarkers. Eur. J. Nucl. Med. Mol. Imaging 2011;39:399–407.

6. Fayad ZA, Mani V, Woodward M, et al. Safety and efficacy of dalcetrapib on atherosclerotic disease using novel non-invasive multimodality imaging (dal-PLAQUE): a randomised clinical trial. Lancet 2011;378:1547–59.

7. Elkhawad M, Rudd JHF, Sarov-Blat L, et al. Effects of p38 mitogen-activated protein kinase inhibition on vascular and systemic inflammation in patients with atherosclerosis. JACC. Cardiovasc. Imaging 2012;5:911–22.

8. Tawakol A, Fayad Z a., Mogg R, et al. Intensification of Statin Therapy Results in a Rapid Reduction in Atherosclerotic Inflammation: Results of A Multi-Center FDG-PET/CT Feasibility Study. J. Am. Coll. Cardiol. 2013;62:909–17.

9. van Wijk DF, Sjouke B, Figueroa A, et al. Nonpharmacological Lipoprotein Apheresis Reduces Arterial Inflammation in Familial Hypercholesterolemia. J. Am. Coll. Cardiol. 2014;64:1418–26.

10. Tawakol A, Singh P, Rudd JHF, et al. Effect of treatment for 12 weeks with rilapladib, a lipoprotein-associated phospholipase A2 inhibitor, on arterial inflammation as assessed with 18F-fluorodeoxyglucose-positron emission tomography imaging. J. Am. Coll. Cardiol. 2014;63:86–8.

11. van der Valk FM, van Wijk DF, Lobatto ME, et al. Prednisolone-containing liposomes accumulate in human atherosclerotic macrophages upon intravenous administration. Nanomedicine 2015;11:1039–1046.

12. Kootte RS, Smits LP, van der Valk FM, et al. Effect of open-label infusion of an apoA-I-containing particle (CER-001) on RCT and artery wall thickness in patients with FHA. J. Lipid Res. 2015;56:703–12.

13. Emami H, Vucic E, Subramanian S, et al. The effect of BMS-582949, a P38 mitogen-activated protein kinase (P38 MAPK) inhibitor on arterial inflammation: A multicenter FDG-PET trial. Atherosclerosis 2015;240:490–6.

14. Gaztanaga J, Farkouh M, Rudd JHF, et al. A phase 2 randomized, double-blind, placebo-controlled study of the effect of VIA-2291, a 5-lipoxygenase inhibitor, on vascular inflammation in patients after an acute coronary syndrome. Atherosclerosis 2015;240:53–60.

**Online Table 2:** Additional arterial wall ^18^F-FDG uptake metrics in study groups

|  | **Healthy control subjects** | **Patients at increased CVD risk** | **Patients with known CVD** | **P-value*** |
| --- | --- | --- | --- | --- |
| **Remote arterial background SUV_mean_** |  |  |  |  |
| Brachiocephalic arteries | 1.04 ± 0.11 | 1.00 ± 0.14 | 1.01 ± 0.17 | ns |
| Descending aorta | 0.96 ± 0.13 | 0.96 ± 0.16 | 1.02 ± 0.21 | ns |
| **Arterial wall SUV – Remote arterial background SUV** |  |  |  |  |
| Carotid arteries | 0.45 ± 0.21 | 0.78 ± 0.24 | 0.88 ± 0.29 | <0.001 |
| Aorta | 1.01 ± 0.20 | 1.34 ± 0.25 | 1.61 ± 0.44 | <0.001 |
| **TBR_mean_**^§^ |  |  |  |  |
| Carotid arteries | 1.37 ± 0.18 | 1.55 ± 0.20 | 1.69 ± 0.29 | <0.001 |
| Aorta | 1.82 ± 0.28 | 2.08 ± 0.30 | 2.20 ± 0.36 | <0.001 |
| **TBR_most diseased segment_**^§^ |  |  |  |  |
| Carotid arteries | 1.63 ± 0.25 | 2.00 ± 0.36 | 2.18 ± 0.35 | <0.001 |
| Aorta | 2.44 ± 0.28 | 2.93 ± 0.44 | 3.07 ± 0.61 | <0.001 |

Multivariate analysis adjusted for age, gender, hypertension, smoking, body mass index, drug usage, lipid profile and glucose. MDS, most diseased segment; TBR, target to background ratio; CVD, cardiovascular disease.

^§^ TBR = arterial wall SUV_max_ / Venous background SUV_mean_.

**Online Table 3.** Active segment analysis in study groups.

|  |  | **Healthy control subjects** | **Patients at increased CVD risk** | **Patients with known CVD** | **P-value*** |
| --- | --- | --- | --- | --- | --- |
| **Carotid arteries** | **Active defined as** |  |  |  |  |
|  | Active defined as ≥1.80 | 26% of subjects | 86% of patients | 93% of patients |  |
|  | %_active slices_ | 14 ± 26% | 64 ± 32% | 78 ± 66% | 0.005 |
|  | TBR_active slices_ | 1.96 ± 0.09 | 2.10 ± 0.27 | 2.19 ± 0.27 | ns |
|  | Active defined as ≥2.20 | 14% of subjects | 63% of patients | 79% of patients |  |
|  | %_active slices_ | 5 ± 14% | 42 ± 41% | 51 ± 40% | 0.044 |
|  | TBR_active slices_ | 2.09 ± 0.07 | 2.27 ± 0.20 | 2.28 ± 0.22 | ns |
| **Ascending aorta** | **Active defined as** |  |  |  |  |
|  | Active defined as ≥2.60 | 76% of subjects | 95% of patients | 96% of patients |  |
|  | %_active slices_ | 75 ± 22% | 80 ± 29% | 83 ± 24% | ns |
|  | TBR_active slices_ | 2.85 ± 0.11 | 3.10 ± 0.31 | 3.10 ± 0.49 | ns |
|  | Active defined as ≥2.80 | 63% of subjects | 74% of patients | 80% of patients |  |
|  | %_active slices_ | 25 ± 33% | 47 ± 48% | 52 ± 46% | ns |
|  | TBR_active slices_ | 2.92 ± 0.08 | 3.17 ± 0.26 | 3.34 ± 0.37 | 0.015 |

* Multivariate analysis adjusted for age, gender, hypertension, smoking, body mass index, drug usage, lipid profile and glucose. TBR, target to background ratio; CVD, cardiovascular disease.

**Online Table 4:** ^18^F-FDG uptake threshold values for a 95% tolerance interval

| **Artery** | **Metric** | **Threshold*** | **Percentage above threshold** | |
| --- | --- | --- | --- | --- |
|  |  |  | Patients at increased CVD risk | Patients with known CVD |
| Carotid | SUV_max_ | > 1.95 | 22% | 43% |
|  | TBR_max_ | > 1.93 | 43% | 43% |
| Aorta | SUV_max_ | > 2.49 | 35% | 51% |
|  | TBR_max_ | > 2.77 | 50% | 62% |

TBR, target to background ratio; CVD, cardiovascular disease.

* Thresholds were determined using the 95^th^ percentile value observed in the healthy control subjects.

**Online Table 5.** Intra- and inter-observer agreement for TBR_max_.

|  | **Carotid arteries** | **Aorta** |
| --- | --- | --- |
| **Intra-observer** |  |  |
| Paired diff between read 1A and 1B | 0.01 ± 0.05 | 0.02 ± 0.05 |
| COV | 2.7% | 1.8% |
| Intra ICC [CI] | 0.99 [0.98 – 1.00] | 0.99 [0.99 – 1.00] |
| **Inter-observer** |  |  |
| Paired diff between read 1A and 2 | 0.03 ± 0.11 | 0.07 ± 0.14 |
| COV | 6.0% | 5.2% |
| Inter ICC [CI] | 0.96 [0.93 – 0.97] | 0.97 [0.96 – 0.99] |

CI, confidence interval; COV, coefficient of variation; diff, difference; ICC, intraclass correlation coefficient; TBR, target to background ratio.

**Online Table 6.** Interscan agreement for ^18^F-FDG uptake metrics.

|  | **Paired difference** | **COV** | **Interscan ICC [CI]** |
| --- | --- | --- | --- |
| Arterial wall SUV | 0.01 ± 0.08 | 3.0% | 0.93 [0.73-0.98] |
| Venous background SUV | 0.05 ± 0.05 | 5.5% | 0.96 [0.81-0.99] |
| Remote arterial background SUV | 0.06 ± 0.05 | 4.9% | 0.93 [0.73-0.98] |
| Arterial wall SUV – Venous background SUV | 0.03 ± 0.06 | 3.5% | 0.93 [0.80-0.98] |
| Arterial wall SUV – Remote arterial background SUV | 0.03 ± 0.07 | 4.3% | 0.95 [0.78-0.94] |
| TBR^§^ | 0.02 ± 0.06 | 2.9% | 0.98 [0.92-1.00] |

CI, confidence interval; COV, coefficient of variation; diff, difference; ICC, intraclass correlation coefficient; TBR, target to background ratio. § TBR = arterial wall SUV_max_ / Venous background SUV_mean_.

**Online Figure 1:** Estimated sample sizes for vascular intervention studies using SUV_max_

Sample sizes, dependent on estimated drug effect (5-20%) and target vessel (carotid or aorta), required for studies using SUV_max_ as the primary endpoint are approximately 20-45% higher compared with TBR_max_ as an endpoint.

**Online Figure 2:** Bland-Altman plots for scan and observer agreement

Bland-Altman plots display no fixed or proportional bias for TBR_max_ in interscan, intra- and inter-observer agreement as shown for the carotids (A-C) and aorta (D-F). TBR, target to background ratio.
